# The effect of dietary energy and protein level on feather, skin and nodule growth of the ostrich (*Struthio camelus*)

**DOI:** 10.4102/jsava.v91i0.2000

**Published:** 2020-09-17

**Authors:** Tertuis S. Brand, Werne J. Kritzinger, Daniel A. van der Merwe, Anieka Muller, Johannes P. van der Westhuyzen, Louwrens C. Hoffman

**Affiliations:** 1 Department of Animal Sciences, University of Stellenbosch, Stellenbosch, South Africa; 2 Animal Sciences, Western Cape Department of Agriculture, Elsenburg, South Africa; 3 Centre for Nutrition and Food Sciences, Queensland Alliance for Agriculture and Food Innovation, University of Queensland, Coopers Plains, Australia

**Keywords:** ostrich, energy, protein, feather, nodule, skin, diet

## Abstract

Accurate diet formulations are required to fulfil the nutrient requirements of birds in order to achieve optimal production. Knowing how the skin, nodule and feather production characteristics vary with diets of different nutrient densities will help in least-cost modelling. Feather growth and nodule development are factors that were previously neglected in ostrich diet formulation, both of which are essential for the development of a predictive production model. In this trial, 120 birds were placed in 15 pens. Varying energy regimes (high, medium and low) and accompanying protein and amino acid profile levels (level 1–5) were assigned *ad libitum* to each pen. A randomly selected bird from each pen was slaughtered at 1, 35, 63, 103, 159, 168 and 244 days of age. During the slaughter, each bird was weighed, stunned, exsanguinated, defeathered and eviscerated. Feathers from four regions of the skin were plucked and weighed. The shaft diameter of the wing feathers was measured. The nodule size of the tanned skin was measured for each slaughter age. The data were transformed to natural logarithms and regressed against the total feather weight and the total featherless empty body protein weight to set up allometric growth equations. A prediction equation to determine nodule size of the live bird was proposed. Feed cost optimisation is paramount, and results from this study will aid in setting up least-cost optimisation (simulation) formulation models.

## Introduction

Ostriches are primitive, flightless birds that were originally domesticated to produce feather plumes, which were popular in the fashion industry. Depending on the quality of the product, feathers can potentially contribute up to 40% of the income generated by a bird, with leather and meat also contributing 35% and 25%, respectively (Dr A. Olivier, pers. comm. Ostrich Business Chamber, Oudtshoorn 6620, South Africa, April 2020).

As the leather produced from ostrich skins is marketed as a luxury product, and is sold at a high price compared to the leather of other livestock (Cooper 2001), knowledge of the factors affecting the physical properties is required (Sales 1999). To maximise skin yield for leather production, it is important to optimise nutrition of the birds while also preventing scarring and bruising caused by transporting, pecking, parasites and arthropod infestations (Cooper 2001). Skin nodules are the result of feather follicles that expand around the quill on the ostrich skin. Nodules distinguish ostrich skin from other skins and make it unique in appearance and touch. Although no formal standards are available, the size, shape and distribution of these nodules are expected to have an effect on the marketability of the product (Cloete et al. 2004). Cloete et al. (2004) reported that the age of the bird has an effect on some nodule traits for birds up to 14 months of age. Mellet, Fisher and Bohme (1996) reported that nodule size increases with an increase in age, with an optimum nodule size being achieved only at 14–16 months of age. However, Van Schalkwyk et al. (2001) showed that age, along with body weight, influenced nodule size and that acceptable nodule sizes could be achieved at 11 months of age. The skin of the ostrich also grows tougher with age and therefore skins from younger birds are used for producing more delicate leather products (Cooper 2001). Nodule shape is determined by the stage of feather growth at the time of slaughter (Mellett 1996). Swart 1981 (as cited by Van Schalkwyk 2008) reported that green body feathers or blood feathers lead to inferior nodules. Cooper (2001) also advised a cautious approach for feather removal from a live bird because damage to the follicle could lead to inferior nodule shapes, as round and uniform nodules are appealing to the customers. Large, deep-bodied birds with an abundance of feathers produce the most desirable skins and when marketed generate the highest premiums (Sales 1999). The combination of the above-mentioned factors on the growth and development of nodule size and shape is a topic of much discussion and deliberation. Because it is known that the nutrition level affects body weight gain (Brand et al. 2000, 2003; Swart & Kemm 1985), investigating the nutrition level’s influence on nodule size is of paramount importance. Angel (1996) identified factors that could cause skin damage. Among these factors, the most important factor, relating to nutrition, is that excess fat accumulation under the skin renders the salt and bacterial treatment of skins after slaughter ineffective.

The body grows and develops in a systematic manner. Therefore, it is expected that the requirements for dietary nutrients in growing animals will also be changing systematically (Bradford & Gous 1991). Feather protein and body protein differ in their respective amino acid compositions (Emmans 1989). The proportion of feather protein, with respect to the total protein content, changes as the bird grows and develops. Therefore, this needs to be taken into account when determining amino acid requirements of the birds (Emmans 1989). Quilt mite and lice infestations, especially on the femina feathers, in ostriches are quite common, and by biting and chewing the feathers the yield as well as quality of the feathers can be compromised (Cooper & El Doumani 2006). However, very little data are available on feather growth as well as factors that influence the growth thereof in ostriches. A starting point for such calculations is an adequate description of feather growth (Gous et al. 1999). Knowing how the skin and feathers develop and how changes in dietary nutrients affect this is necessary for an accurate assessment of nutrient requirement.

This study was conducted to investigate the effect of dietary energy and protein level on various skin and feather traits, as well as identifying the various factors affecting nodule and feather growth from the first day of life up to trial termination (244 days old).

## Materials and methods

Overall, 120 birds were randomly allocated to 15 pens (eight birds per pen). Varying energy regimes (high, medium and low) and accompanying protein (amino acid) levels (level 1–5) were formulated (Table 1) for the birds in each of the pens. Four phases (pre-starter, starter, grower and finisher) of feeding were used, and feed was supplied *ad libitum* throughout the trial. The energy regimes consisted of three increasing levels of dietary energy and similarly increasing protein levels to give a total of 15 diets per growth phase (Table 1). The median feeding regime was chosen according to standard requirement levels for energy and protein (amino acids) from literature (Du Preez 1991; Smith et al. 1995 and Cilliers 1998, as summarised by Brand 2008). The diets were formulated with maize meal, wheat bran, soybean meal and Lucerne hay (Table 2) as base ingredients in order to meet the requirements of the growing birds (Brand 2008). From the diet formulated with respect to the standard requirements (median feeding regime), the proportions of the ingredients were adjusted in order to reduce or increase the dietary energy by 20% and dietary protein by 10% and 20%, respectively, in each feeding phase (Table 1). An average feed intake per bird (Table 3) was determined by weighing the supplied feed and the feed leftovers for each pen throughout each phase. The feeds were sampled and analysed (Association of Official Analytical Chemists methods 2002) for protein, amino acids, fat, neutral detergent fibre (NDF), acid detergent fibre (ADF), ash and fibre. Water was available *ad libitum* throughout the trial. During the study, a randomly selected bird from each pen (treatment group) was slaughtered at 1, 35, 63, 103, 159, 168 and 244 days of age.

**TABLE 1:** A representation of the energy and protein levels for the 15 feeding regimes with varying levels of energy and protein in the diet, formulated for the pre-starter, starter, grower and finisher phases of ostrich chicks.

Diet regime	Energy level (MJ/kg feed)			Protein levels (g/kg feed)				
	Low	Medium	High	1	2	3	4	5
Pre-starter	12.5	14.5	16.5	170	190	210	230	250
Starter	10.5	12.5	14.5	140	160	180	200	220
Grower	8.5	10.5	12.5	115	135	155	175	195
Finisher	7.5	9.5	11.5	80	100	120	140	160

**TABLE 2:** Representation of the raw material inclusions for the medium energy and medium protein inclusions for each growth phase. These are the standard requirements for energy and protein.

Ingredients (g/kg)	Pre-starter	Starter	Grower	Finisher
Maize meal	343	273	-	79
Oat bran	-	75	291	361
Barley meal	-	75	288	165
Lucerne meal	50	330	179	215
Wheat bran	250	9	53	33
Sunflower oil-cake meal	-	-	-	50
Soya-bean oil-cake meal	119	39	94	32
Full-fat soya meal	130	80	1.3	17
Fish meal	50	76	44	8
Plant oil	21	13	5	5
Synthetic lysine	0.4	0.3	0.7	0.7
Synthetic methionine	1.5	0.9	11	0.9
Mono-calcium phosphate	2.1	9	2	-
Di-calcium phosphate	-	-	8	13
Limestone	27	11	14	13
Vitamin and mineral mix	2.5	50	50	50
Salt	4	4	4	4

**TABLE 3:** A description of the average feed intake for the feeding regimes, with varying energy and protein levels provided to ostriches during the pre-starter, starter, grower and finisher phases.

Feeding regime	Energy level	Protein level	Feed intake (g/bird/day)			
			Pre-starter (0–1 months)	Starter (1–3 months)	Grower (3–6 months)	Finisher (6–9 months)
1	Low	1	392	556	2526	4778
2	Low	2	421	907	2581	4356
3	Low	3	445	796	2577	4217
4	Low	4	353	873	2530	4001
5	Low	5	384	723	2959	4038
6	Medium	1	433	539	2210	3854
7	Medium	2	489	612	2162	3447
8	Medium	3	209	742	2264	2997
9	Medium	4	473	486	2249	3808
10	Medium	5	228	531	2690	3602
11	High	1	195	581	2292	3210
12	High	2	212	486	2131	2909
13	High	3	162	438	2191	2992
14	High	4	133	891	2004	2815
15	High	5	161	464	1710	2773

At each prescribed slaughter age, birds were weighed, stunned, exsanguinated, defeathered and eviscerated. During exsanguination, the blood of each bird was collected in a separate container. Feathers were plucked from the wings (white plumes), byocks, tail and drab floss body regions and dried at 80 °C for 48 hours in a drying oven. The feathers were then weighed separately according to the body region and as a total yield. The shafts of 10 randomly selected wing feathers (five on each side) were measured at the base (point of skin entry) using a digital calliper. After feather removal from the carcass, the skin was flayed, weighed and the surface area was determined by spreading the wet skin over a linen cloth and by tracing the outlines. The traced version was cut out, and the surface area was determined using computerised video image analysis. Ten randomly selected nodules per skin region were then measured, using a digital calliper, on the tail, median and flank regions of the skin.

The intestines of each bird were cleaned with water and weighed. The heart and liver were removed and weighed. The body as a whole, together with the clean intestines, heart, liver and feathers were minced and mixed thoroughly after which one randomly selected sample (approximately 150 g) was used to perform a proximate analysis (AOAC methods 2002), yielding the chemical composition of the entire bird. The feathers were analysed together with the remainder of the body. After the analysis of ostrich feathers was obtained in a separate study (Kritzinger 2010), the proportional protein contribution of the feathers was deducted from the protein analysis obtained above, thus yielding an estimation of the featherless empty body protein weight (EBPW) and was used for further statistical analyses. Emmans (1989) reported that feather protein and body protein should not be analysed together. Swart, Mackie and Hayes (1993a) observed considerable quantities of gut fill (8% – 15% of live weight), and noted that major variation can occur between individuals at any specific time. This motivated the use of empty gut weight rather than live weight.

As growth is non-linear (Huxley 1932; Lawrie 1998; McDonald et al. 2002), all the data were transformed into the natural logarithmic form in order to obtain linear growth data. Using the Mixed Models Procedure of Statistical Analysis System (SAS) statistical software version 9.1 (SAS Institute Inc., Cary, NC, United States), the data were analysed for differences between birds on different treatments by comparing the slopes and intercepts independently. In the cases where no differences were found between the treatments, a general regression line was fitted to the data. This provided an equation to compare and predict body component growth by utilising the existing allometric relationships between components and EBPW. No differences were found between dietary protein levels for all the investigated variables, and these results are not reported. A principal component factor analysis was performed using Statistica, version 8.0 (StatSoft Inc. 2008), to determine the effect of certain explanatory variables on the average nodule size of the ostrich skin.

### Ethical consideration

Ethical clearance was obtained from the Department Ethical Committee for Research on Animals (DECRA), Western Cape Government (project number R10/13).

## Results

The energy and protein levels used to formulate the diets at each respective growth phase in this study are presented in Table 1. A description of average feed intake per bird per day of each feeding regime at the growth phase is presented in Table 3. It is evident that the average feed intake increases with the growth phase, as can be expected with increase in size of the ostrich chicks (Brand et al., 2018b, 2019). The energy level of the diet was found to influence the average intake in the pre-starter, grower and finisher phases, with feed intake decreasing from the low- to high-energy regimes (*p* < 0.05). Conversely, the level of dietary protein did not influence the average feed intake at any of the growth phases (*p* > 0.05).

The EBPW of the ostriches was regressed over the slaughter ages and analysed according to the dietary energy and protein levels. Linear regression coefficients for the intercept and slope of the curves are presented in Table 4. As the birds had not yet reached the plateau phase of the growth curve where they attain mature body weights (Brand et al. 2019), EBPW still increased in a linear fashion, with no indication of attaining a mature EBPW. The level of energy in the diet influenced the value of the intercept as well as the slope of the curves for the increase in EBPW (*p* < 0.05). The curve for the low energy level displayed the lowest intercept (-0.752 ± 0.125) and the greatest slope value (0.178 ± 0.006) than the medium- and high-energy level diets. This indicates that the EBPW of ostrich chicks reared on lower energy levels increased at a faster rate than that of the birds reared on higher energy levels. The protein levels of the diets, on the other hand, did not significantly influence the intercepts or slopes of the curves (*p* < 0.05), indicating that regardless of the protein level in the diet, the EBPW of the birds increases at the same rate.

**TABLE 4:** Linear regression coefficients (±S.E.) for the increase in empty body protein weight (kg) with age of ostrich chicks (days) analysed for increasing dietary energy and protein levels.

Diet composition	Intercept	Slope	*R*^2^
**Energy level**			
Low	−0.752‡ ± 0.125	0.178† ± 0.006	0.96
Medium	−0.357†‡ ± 0.125	0.154‡ ± 0.006	0.98
High	−0.260† ± 0.125	0.146‡ ± 0.006	0.94
**Protein level**			
1	−0.423 ± 0.225	0.149 ± 0.012	0.96
2	−0.546 ± 0.225	0.160 ± 0.012	0.93
3	−0.550 ± 0.225	0.160 ± 0.012	0.94
4	−0.417 ± 0.225	0.164 ± 0.012	0.95
5	−0.343 ± 0.225	0.165 ± 0.012	0.97

†, ‡ , Values with different superscripts in the same column differ significantly (*p* < 0.05)

The effect of the dietary energy regimes on the wet skin size and the wet skin weight of the ostrich is depicted in Table 5. It is evident that skin sizes and skin weights differed (*p* < 0.05) between the energy regimes, with the low-energy diet having the smallest skin size (2.63 ± 0.14) and the lightest skin weight (-1.44 ± 0.18) and the high-energy diet having the largest skin size (3.60 ± 0.10) and the heaviest skin weight (-0.13 ± 0.13). To increase the accuracy of the results, the least square means were included as these results are adjusted for differences that might occur between the intercepts. Comparison of the least square means (Table 6) indicated significant differences between the intercepts of the analysed data. An increase in the energy density of the diet had a positive (*p* < 0.05) effect on the skin weight (kg) and the skin size (square decimetre [dm^2^]) of the ostriches (Tables 5 and 6).

**TABLE 5:** Allometric equations relating the natural logarithm of the skin characteristics to the natural logarithm of the empty body protein weight (kg) for each energy level.

Variable	Energy level	Skin size (dm^2^)	Skin weight (kg)
Constant term	Low	2.63† ± 0.14	−1.44† ± 0.18
	Medium	3.21‡ ± 0.14	−0.66‡ ± 0.18
	High	3.60§ ± 0.10	−0.13§ ± 0.13
Regression coefficient	Low	0.30† ± 0.07	0.32† ± 0.10
	Medium	0.39†‡ ± 0.07	0.50†‡ ± 0.09
	High	0.47‡ ± 0.05	0.62‡ ± 0.07
*R*^2^	-	0.72	0.72

†, ‡, § , Values with different superscripts in the same column, differ significantly (*p* < 0.05)

**TABLE 6:** A comparison of the least square means (±SE) of the natural logarithms of the slope of skin size and weight regressed against the natural logarithm of empty body protein weight as adjusted for intercept.

Energy level	Low	Medium	High	*R*^2^
Skin size (dm^2^)	2.953† ± 0.084	3.639‡ ± 0.081	4.117§ ± 0.080	0.72
Skin weight (kg)	−1.086† ± 0.110	−0.107‡ ± 0.106	0.549§ ± 0.104	0.72

†, ‡, § , Values with different superscripts in the same row, differ significantly (*p* < 0.05).

Allometric growth coefficients of the different feather groups, after evaluation for treatment differences, were calculated (Table 7). No differences (*p* > 0.05) were found between the energy treatments for either the constant term or the regression coefficient. The natural logarithm of the feather weights at each region were regressed against the natural logarithm of the total feather weight in Table 7. The maturation rate of feathers from different regions and the protein weight of the empty body differed and justified the separate prediction of these two variables.

**TABLE 7:** Allometric coefficients relating the natural logarithms of the weighed feathers from different regions to the natural logarithms of the total feather weight.

Feathers†	Constant term	Regression coefficient	*R*^2^
Drab floss	−7.7412 ± 0.1996	1.5914 ± 0.0351	0.95
Tail	−4.5449 ± 0.1631	1.3239 ± 0.0287	0.95
Wing	−3.4884 ± 0.1551	1.3577 ± 0.0273	0.96
Byocks	−5.9769 ± 0.2679	1.6250 ± 0.0471	0.91

† , No differences were found between treatments.

The effect of nodule size (diameter, mm), as measured on the different regions of the skin, and the average nodule size of the corresponding skin at different slaughter ages are compared in Table 8. Nutrient density (energy or protein level) had no effect (*p* > 0.05) on the nodule sizes of the different regions or on the average nodule size. No differences (*p* > 0.05) were found between the nodule sizes of the different regions; this may be because of relatively large intra-group variation; therefore, the data in Table 8 are reported per age group. From Table 8, a general increase in nodule size with an increase in age is notable. Nodules are the largest at age 159 days (3.42 ± 0.06 mm) and 244 days (3.45 ± 0.06 mm) for all the regions on average, and the smallest nodule size was found on birds of aged 1 day (1.22 ± 0.09 mm), followed by those aged 35 days (1.65 ± 0.06 mm). Because energy and protein levels did not influence the quality traits tested in this study, other causative growth factors were investigated for correlations.

**TABLE 8:** Average nodule size (mm) and the standard error of the measured nodules for every slaughter age.

Age (days)	Nodule size (mm)			
	Flank	Median	Tail	Average
1	1.14‡‡ ± 0.01	1.29‡‡ ± 0.15	1.22†† ± 0.09	1.22†† ± 0.09
35	1.63†† ± 0.08	1.65†† ± 0.22	1.61¶ ± 0.08	1.65†† ± 0.06
63	2.12¶ ± 0.08	2.18¶ ± 0.08	2.15§ ± 0.08	2.15§ ± 0.06
103	2.94§ ± 0.08	3.01‡ ± 0.08	3.06‡ ± 0.08	3.00‡ ± 0.06
159	3.38† ± 0.08	3.32†‡ ± 0.08	3.55† ± 0.08	3.42† ± 0.06
168	2.98‡§ ± 0.08	2.60§ ± 0.24	3.06‡ ± 0.08	2.88^¶^ ± 0.06
244	3.30†‡ ± 0.08	3.38† ± 0.08	3.66† ± 0.08	3.45† ± 0.06

†, ‡, §, ¶, †† , Values with different superscripts in the same column, differ significantly (*p* < 0.05).

To understand the correlations among the explanatory variables (Table 9), a principal component factor analysis was calculated. Age, live weight, EBPW, shaft diameter, skin size, skin weight and total feather weight were transformed to the natural logarithmic form and analysed. All the explanatory variables, except shaft diameter and total feather weight, were co-linear and loaded onto the first factor (Table 10). Only two factors were found to influence the nodule size (Table 10). The second factor loads were observed only on the wing feather shaft diameter and the total feather weight. When constructing a regression model following a factor analysis, the prediction variables should preferably come from different factors to avoid the problem of co-linearity. A multiple linear regression model, with the independent variables live weight and shaft diameter, was firstly constructed (explaining 83% variation) and then improved with response surface regression to include a quadratic term, thus yielding the equation:

$$\begin{array}{l} y=1.258943+0.047555x-0.000375x^{2}+0.130603z\\ (R^{2}=0.85), \end{array}$$ [Eqn 1]

where *y* = nodule size (mm), *x* = live weight (kg), and *z* = shaft diameter (mm).

**TABLE 9:** Correlation coefficients (r) between the measured explanatory variables affecting nodule size. All correlations are significant at *p* < 0.05.

Variable	Correlationscase-wise *N* = 100							
	EBPW (kg)	Live weight (kg)	Age (days)	Nodule size (mm)	Total feathers (g)	Shaft diameter (mm)	Skin weight (kg)	Skin size (dm^2^)
EBPW (kg)	1.00	0.97	0.96	0.84	0.87	0.81	0.90	0.93
Live weight (kg)	-	1.00	0.96	0.81	0.86	0.78	0.97	0.95
Age (days)	-	-	1.00	0.88	0.92	0.89	0.92	0.97
Nodule size (mm)	-	-	-	1.00	0.83	0.91	0.77	0.85
Total feathers (g)	-	-	-	-	1.00	0.89	0.80	0.93
Shaft diameter (mm)	-	-	-	-	-	1.00	0.71	0.87
Skin weight (kg)	-	-	-	-	-	-	1.00	0.92
Skin size (dm^2^)	-	-	-	-	-	-	-	1.00

EBPW, empty body protein weight.

**TABLE 10:** Individual factor loadings as determined by factor analysis (significant contributions for each factor are in bold type).

Variable	Factor 1	Factor 2
EBPW (kg)	**0.721574**	0.461219
Live weight (kg)	**0.836427**	0.417353
Total feathers (g)	0.478441	0.638494
Shaft diameter (mm)	0.385094	**0.887545**
Skin weight (kg)	**0.906954**	0.345921
Skin size (dm^2^)	**0.705680**	0.572084
Age (days)	**0.707225**	0.598385
Explanatory variable	3.418199	2.387322
Proportion of total	0.488314	0.341046

Note: Values in bold are significant at *p* < 0.05.

EBPW, empty body protein weight.

A multiple linear regression model, with the independent variables age and shaft diameter, was also constructed and yielded the following equation:

$$y=1.179158+0.001674x+0.318662z\;(R^{2}=0.82),$$ [Eqn 2]

where *y* = nodule size (mm), *x* = age (days), and *z* = shaft diameter (mm).

**FIGURE 1:**
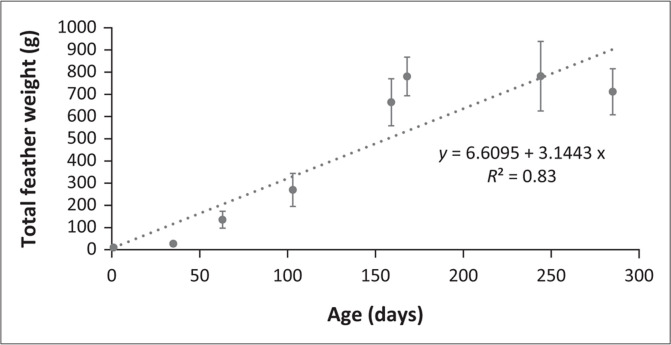
The total feather weight (standard deviation bars) of ostriches with an increase in age.

**FIGURE 2:**
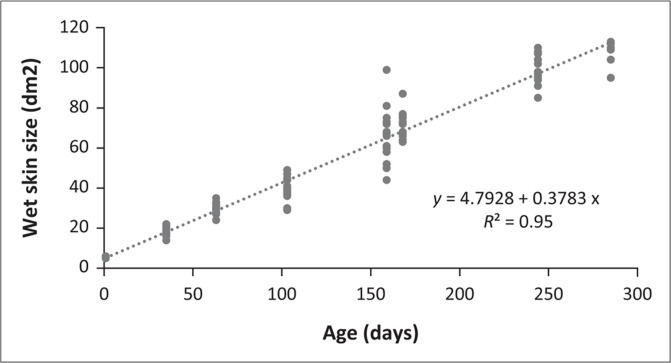
Illustration of the relationship between skin size (dm2) and age (days) in ostriches.

Although the birds received different dietary treatments, all results are included as the general trends and approximate sizes will still be applicable from these data. From the evaluation of growth pattern of feathers (Figure 1), it is evident that the total feather weight increases linearly by 6.6 g per day. Similarly, when examining the relationship between wet skin size and age (Figure 2), wet skin size was seen to increase at a rate of 0.38 dm^2^ per day.

## Discussion

When evaluating average feed intake (Table 3), it is evident that feed intake decreased for higher energy diets. This is supported by the popular belief that monogastric animals will eat to fulfil their energy requirements (McDonald et al. 2002; Schinckel & De Lange 1996). Brand et al. (2018b) also demonstrated the relationship of intake in ostriches decreasing in response to higher dietary energy densities. The higher rates of increase in EBPW in ostriches reared on low-energy diets, along with the higher intakes, suggest that the birds consumed greater quantities of protein, which can be used for enhanced growth. As the protein level did not influence average intake, it did not show any marked effect on the EBPW of the ostriches (Tables 3 and 4). As a result, it can be confirmed that the energy content of the diet influences the level of feed intake in ostriches, which in turn affects the growth of the ostrich chicks. In this study, it was not evident that the level of dietary protein influenced intake, and as a result growth, Brand et al. (2019) observed that intake and growth do increase at higher dietary protein levels. The possibility that the birds will eat to satisfy the requirement of the most limiting nutrient (either energy or amino acid) should therefore not be ruled out yet (Ferguson 2006).

Data from this study indicate that dietary energy level had a significant effect on the skin weight and skin size of ostriches (Table 4 and 5). This coincides with the literature in which Van Schalkwyk et al. (2002) and Brand, Nel and Van Schalkwyk (2000; Brand et al. 2004, 2005) reported that ostriches consuming high-energy concentrations produced skins with a larger surface area than birds consuming lower energy diets. Cloete et al. (2006) reported heavier raw skin weights in ostriches consuming higher energy density diets than those on lower dietary energy levels. The increase in the size and weight of the skin with an increase in dietary energy level could be ascribed to the increase in total body fat of the birds, as they will consume more nutrients than they require. It is known that ostriches deposit large amounts of fat both in the body cavity and subcutaneously (under the skin), the latter being concentrated in specific subcutaneous fat deposits (Brand et al. 2004; Cloete et al. 2006; Swart, Mackie & Hayes 1993b). Brand et al. (2000, 2004, 2005) and Cloete et al. (2006) reported that dietary protein failed to influence the measured skin characteristics. One must also bear in mind that an increase in the mass of any animal will result in a larger surface area of the body. The larger surface area alone may be responsible for the increased size and mass of the skin. Therefore, the nutrient density does not have a direct influence on skin traits but does influence the growth of the birds, which in turn impacts on the size and mass of the skins.

Although the linear equation in Figure 1 gives a relatively good fit (*R*^2^ = 0.83), it is possible that feather growth is non-linear and peaks before plateauing out on the graph beyond the age of 150 days. Table 6 confirms that the maturation rate of feather growth and the protein weight of the empty body differs; thus, defining feather growth separately from EBPW is of utmost importance as the two differ in growth rate, as suggested by literature (Emmans 1989; Emmans & Fisher 1986). The amino acid composition of feathers is different from the amino acid composition of body protein (Emmans 1989). The feather growth per region in relation to total feather growth of the current study compares favourably with similar results on one diet (Brand et al. 2018a). This is a confirmation of results in this study, namely, that diet does not significantly affect feather growth.

The linear relationship between the increase in skin size of all the birds and age (Figure 2) is because of the fact that it was measured over a short interval and not up to the point where birds attain mature body weights. Generally, it is accepted that biological tissues, including ostrich skin, will follow a non-linear growth pattern (Brand et al. 2018a; Huxley 1932; McDonald et al. 2002), but this will only become evident over extended periods of growth as the birds pass maturity (Mellet 1992). The fact that the skin size increases linearly for the first 244 days of the growth cycle can be a useful prediction instrument for slaughter birds and is in accordance with reports from Van Schalkwyk et al. (2002), as they reported a linear increase in skin area with an increase in age. Mellet et al. (1996) stated that the industry required a skin size of 120 dm^2^, which could be obtained at the age of 10 months. When converted from the wet skin weight to the dry skin weight (*y* = 0.791587*x* + 52.31, *R*^2^ = 0.64; Brand 2008 – unpublished results); results from this study confirm this observation, as the equation predicts a skin size of 136.8 ± 5.0 dm^2^ for the 285-day-old birds. Van Schalkwyk et al. (2001) indicated that protein levels had no effect on nodule development; similar results are reported in this study as well (Table 7).

Mellet et al. (1996) reported slaughter age or degree of maturity as the primary factor driving leather quality, and that the optimum nodule size is only achievable at the age of 14–16 months. However, Van Schalkwyk (2008) reported that birds can be slaughtered at 11–12 months of age as a result of improved feeding regimes. This was confirmed by Cloete et al. (2004) where the acceptable nodule size and shape could be attained at 11 months of age. The current study identified several factors that affect nodule development. All these factors are co-linear (Table 8), which complicates the interpretation of the results. Practical variables that describe model variation sufficiently were used to construct the prediction model for this study. The model allows the prediction of the nodule size up to an age of 285 days (*R*^2^ = 0.85). The average nodule size can now be estimated while the bird is still alive, and this will aid in least-cost simulation modelling by determining the live weight (irrespective of gut content) and the feather shaft diameter.

## Conclusion

The effects of different dietary energy and protein levels on the various skin and feather characteristics were investigated, while feather and nodule growths were also described. Higher dietary energy levels were seen to increase the skin yield, but increasing dietary protein levels had no effect. A prediction equation that enables the determination of nodule size directly from the live bird’s age was derived. Simulation modelling will increase the effectiveness and usefulness of the results in this study, as the combining effect will aid in optimising feed costs and add to the ability of the ostrich industry to adapt to challenging and changing economic environments.
